# Synthesis and crystal structure of a cadmium(II) coordination polymer based on 4,4′-(1*H*-1,2,4-triazole-3,5-di­yl)dibenzoate

**DOI:** 10.1107/S2056989024000185

**Published:** 2024-01-09

**Authors:** Anastasiia M. Popovych, Liudmyla V. Tsymbal, Dmytro M. Khomenko, Alexandra Bargan, Yaroslaw D. Lampeka, Rostislav D. Lampeka

**Affiliations:** a L. V. Pisarzhevskii Institute of Physical Chemistry of the National Academy of Sciences of Ukraine, Prospekt Nauki 31, 03028, Kyiv, Ukraine; b Department of Chemistry, Taras Shevchenko National University of Kyiv, Pavlo Skoropadskyi st., 12, Kyiv 01033, Ukraine; c Enamine Ltd., Winston Churchill st., 78, Kyiv 02094, Ukraine; d "Petru Poni" Institute of Macromolecular Chemistry, Aleea Grigore Ghika Voda 41A, RO-700487 Iasi, Romania; University of Aberdeen, United Kingdom

**Keywords:** crystal structure, coordination polymer, cadmium, 4,4′-(1*H*-1,2,4-triazole-3,5-di­yl)dibenzoate, π-stacking, hydrogen bonds

## Abstract

The asymmetric unit of the title compound, *catena*-poly[[[aqua­bis­(pyridine-κ*N*)cadmium(II)]-μ_2_-4,4′-(1*H*-1,2,4-triazole-3,5-di­yl)dibenzoato-κ^4^ *O*,*O*′:*O*′′,*O*′′′] 4.5-hydrate], {[Cd(C_16_H_9_N_3_O_4_)(C_5_H_5_N)_2_(H_2_O)]·4.5H_2_O}_*n*_ or {[Cd(bct)(py)_2_(H_2_O)]·4.5H_2_O}_*n*_ (**I**), consists of a Cd^2+^ cation coordinated to one bct^2–^ carboxyl­ate dianion, two mol­ecules of pyridine and a water mol­ecule as well as four and a half water mol­ecules of crystallization. The metal ion in **I** possesses a penta­gonal–bipyramidal environment with the four O atoms of the two bidentately coordinated carboxyl­ate groups and the N atom of a pyridine mol­ecule forming the O_4_N equatorial plane, while the N atom of another pyridine ligand and the O atom of the water mol­ecule occupy the axial positions. The bct^2–^ bridging ligand connects two metal ions *via* its carb­oxy­lic groups, resulting in the formation of a parallel linear polymeric chain running along the [1  1] direction. The coordinated water mol­ecule of one chain forms a strong O—H⋯O hydrogen bond with the carboxyl­ate O atom of a neighboring chain, leading to the formation of double chains with a closest distance of 5.425 (7) Å between the cadmium ions belonging to different chains. Aromatic π–π stacking inter­actions between the benzene fragments of the anions as well as between the coordinated pyridine mol­ecules belonging to different chains results in the formation of sheets oriented parallel to the (  01) plane. As a result of hydrogen-bonding inter­actions involving the water mol­ecules of crystallization, the sheets are joined together in a three-dimensional network.

## Abstract 2

The coordination polyhedron of the Cd^2+^ ion in the title compound is a CdN_2_O_5_ penta­gonal bipyramid formed by two bidentately coordinated carb­oxy­lic groups of different anions, one water mol­ecule and two pyridine mol­ecules. In the crystal, polymeric chains propagate along the [1 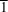 1] direction; the chains are linked by hydrogen bonds and π–π stacking inter­actions into a three-dimensional network.

## 1. Chemical context

Crystalline coordination polymers with permanent porosity (metal–organic frameworks, MOFs) attract much current attention due to the possibilities of their applications in different areas, including gas storage, separation, sensing, catalysis, *etc*. (MacGillivray & Lukehart, 2014 ▸; Kaskel, 2016 ▸). Oligo­carboxyl­ate ligands have become the most popular organic bridging units in MOFs because of their strong coord­ination ability, rich coordination modes and different deprotonation degrees (Rao *et al.*, 2004 ▸; Yoshinari & Konno, 2023 ▸). To a lesser extent, heterocyclic ligands containing several N atoms, which are able to coordinate directly to metal ions, are also used in the construction of MOFs (Chen *et al.*, 2014 ▸; Zhao *et al.*, 2022 ▸). At the same time, hybrid bridging mol­ecules containing both carboxyl­ate functional groups and N-heterocyclic fragment(s) have been studied to a lesser extent (Lu *et al.*, 2023 ▸), although one might expect that the combination of different donor groups in one ligand mol­ecule could open new possibilities for creation of MOFs with specific chemical and structural features.

4,4′-(1*H*-1,2,4-Triazole-3,5-di­yl)di­benzoic acid (H_2_bct; C_16_H_11_N_3_O_4_), a rigid V-shaped ligand possessing two carb­oxy­lic acid groups in symmetrical positions and a N-donor triazole group, belongs to such bridges and is an excellent candidate for the preparation of functional coordination polymers because of several features. It possesses seven potential coordination sites, can adopt various coordination modes due to possible free rotation around C—C bonds between the benzene and the triazole rings, and can partially or completely deprotonate, acting both as a hydrogen-bond acceptor and donor.

The coordination polymers of different metal ions formed by this bridging ligand have been prepared and shown to possess prospective properties including absorption of methane (Li *et al.*, 2022 ▸), catalysis of CO_2_ cyclo­addition reactions (Sun *et al.*, 2019 ▸; Tian *et al.*, 2021 ▸), photocatalysis of dyes degradation (Gao *et al.*, 2023 ▸) *etc*. It has also been shown that this ligand itself demonstrates luminescent properties and its complexes of metal ions with *d* ^10^ electronic configuration (Zn^II^, Cd^II^) or lanthanides can be used as luminescent sensors for different analytes (Zhang *et al.*, 2019 ▸; Luo *et al.*, 2022 ▸; Wang *et al.*, 2022 ▸).

Several coordination polymers formed by the deprotonated bct^2–^ ligand and the Cd^2+^ cation have been described to date and they all possess very similar structures featuring a μ_3_- or μ_4_-bridging mode of the carboxyl­ate (see *Database survey*). The present work describes the preparation and structural characterization of a representative of another type of Cd^II^ coordination polymer, namely, *catena*-poly[[[aqua­bis­(pyri­dine-κ*N*)cadmium(II)]-μ_2_-4,4′-(1*H*-1,2,4-triazole-3,5-di­yl)dibenzoato-κ^4^ *O*,*O*′:*O*′′,*O*′′′] 4.5-hydrate], {[Cd(C_16_H_9_N_3_O_4_)(C_5_H_5_N)_2_(H_2_O)]·4.5 H_2_O}_*n*_, **I**. 
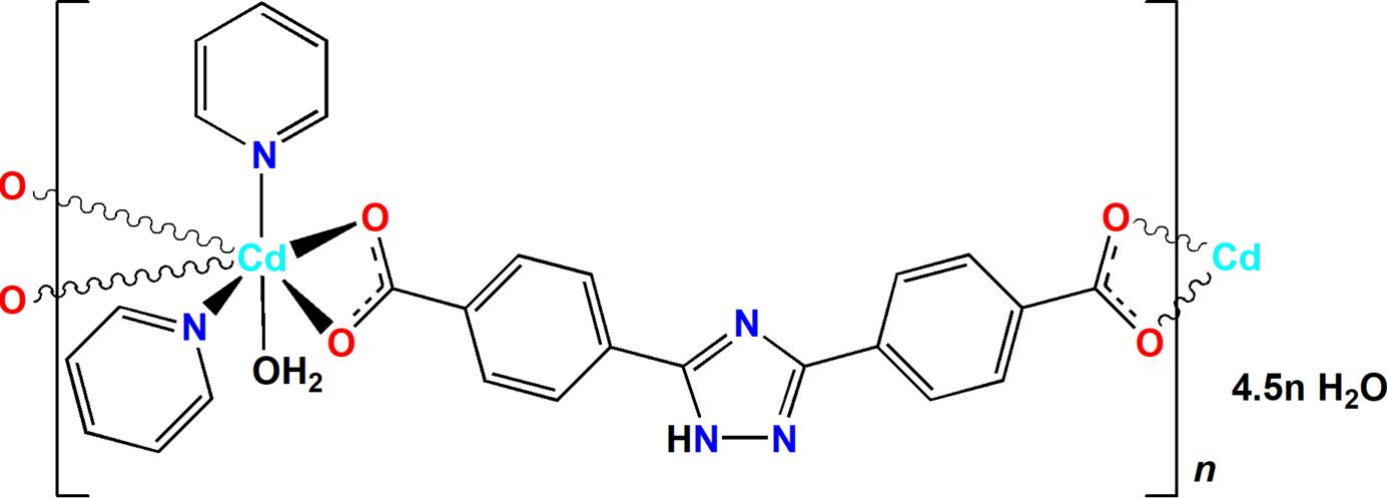


## 2. Structural commentary

**Figure 1:**
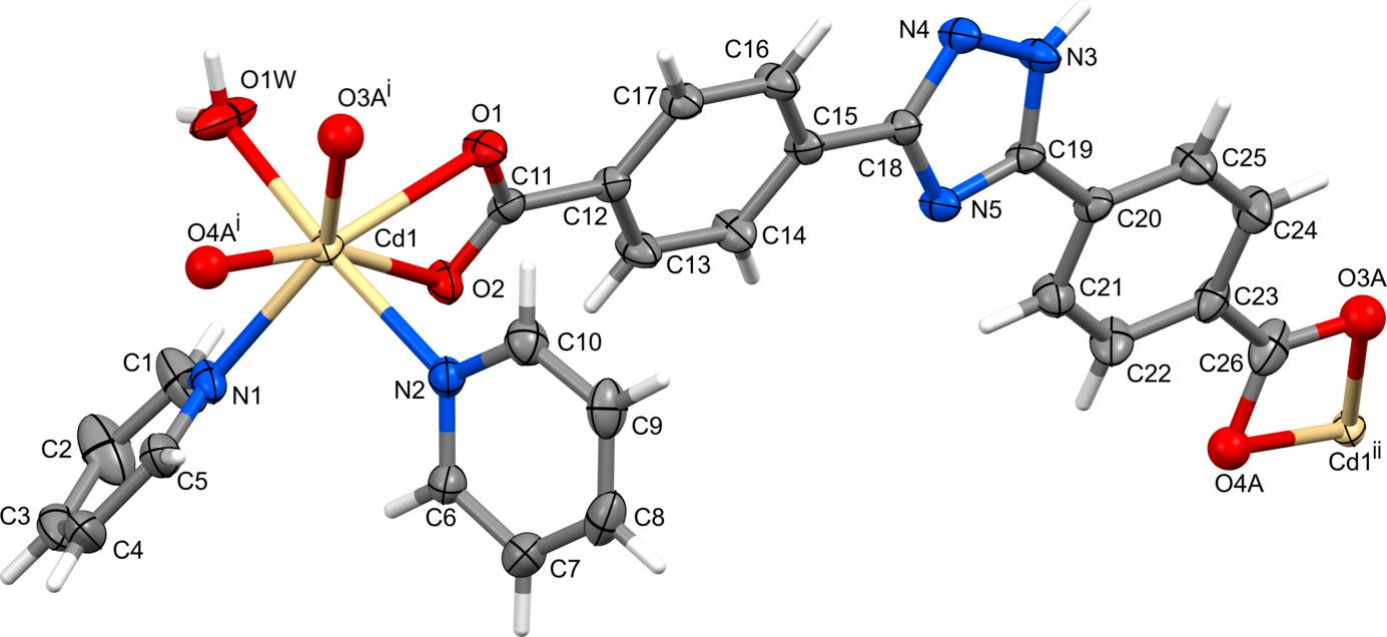
The extended asymmetric unit in **I** showing the coordination environment of the Cd atom and the partial atom-labeling scheme (displacement ellipsoids are drawn at the 30% probability level). The minor occupancy components *B* of the disordered carb­oxy­lic group and water mol­ecules of crystallization are not shown. Symmetry codes: (i) *x* + 1, *y* − 1, *z* + 1; (ii) *x* − 1, *y* + 1, *z* − 1.

The asymmetric unit of complex **I** contains a Cd^II^ cation coordinated to one doubly deprotonated bct^2–^ anion, two mol­ecules of pyridine and a water mol­ecule (Fig. 1 ▸) and includes additionally five water mol­ecules of crystallization, one of which (O6*W*) is disordered over two positions with an occupancy of 0.25 (total of 4.5 water mol­ecules of crystallization). Additionally, one carboxyl­ate group of the anion (C26/O3/O4) is disordered over two orientations with half-occupancy (indices *A* and *B* in the atom-labeling scheme) and these components were refined in an isotropic approximation.

The coordination number of the Cd^II^ ion in **I** is seven and its coordination polyhedron is formed by the two bidentately coordinated carb­oxy­lic groups of different bct^2–^ anions, two pyridine mol­ecules and one water mol­ecule. The metal ion possesses a penta­gonal–bipyramidal environment with the carboxyl­ate O atoms and the N1 atom of pyridine forming the O_4_N equatorial plane, while the N2 atom of another pyridine ligand and O1*W* atom of the water mol­ecule occupy the axial positions. The sum of the angles *D*—Cd—*D* (*D* = donor atom) in the O_4_N equatorial plane is very close to 360° (the difference does not exceed 0.6°), thus evidencing its nearly planar structure and agrees well with a small deviation of the Cd^II^ cation (*ca* 0.09 Å). The orientation of the axial bonds is nearly orthogonal to the equatorial plane (Table 1 ▸). The dihedral angle between pyridine rings is 62.5 (2)°.

The Cd—N bond lengths in **I** are very similar to the Cd—O1*W* distance (*ca* 2.3 Å) and do not depend on the position of the pyridine mol­ecule in the coordination sphere (equatorial or axial). The coordination bonds to these neutral ligands are shorter than those to the majority of O atoms of deprotonated carboxyl­ate groups which, in turn, are significantly non-equivalent within each carboxyl­ate group (Table 1 ▸).

The near equality of the C—O bond lengths in the C11/O1/O2 fragment [1.255 (4) and 1.254 (4) Å] indicate complete electronic delocalization of this carboxyl­ate group. However, this is not the case for both disordered components of the C26/O3/O4 fragment where one C—O bond is significantly shorter than another [*cf*. 1.2485 (10) / 1.2476 (10) Å for the C26—O3*A*/C26—O4*B* bonds and 1.379 (5)/1.343 (6) Å for the C26—O4*A*/C26—O3*B* bonds] thus evidencing mainly localized single and double bond characters of the bonds. Inter­estingly, in these cases Cd1 forms shorter coordination bonds with the carbonyl O atoms. The chelate bite angles of the four-membered chelate rings are determined by the geometrical parameters of the carboxyl­ate groups and are close to 53° (Table 1 ▸).

In the bct^2–^ anion, the carboxyl­ate groups are twisted away from the attached benzene ring to different extent. Whereas the C12/C11/O1/O2 fragment is nearly coplanar with its aromatic ring (*ca* 1.7°) the angle of rotation of the opposite analogue exceeds 10.6°. The conformation of the carboxyl­ate ligand as a whole approximates to twofold rotation symmetry with dihedral angles between the mean planes of the central triazole and lateral benzene rings of 16.1 (2) and 16.5 (2)°, and between the benzene rings of 3.3 (1)°. Inter­estingly, the conformation of the bct^2–^ anion in its disodium salt is notably less planar with angles between the triazole and benzene rings of 14.2 and 28.5° and between the benzene rings of 16.4° (Lu *et al.*, 2021 ▸). Each carboxyl­ate group of the bct^2–^ anion in **I** connects two metal ions and each metal ion is bidentately coordinated by two different anions, thus resulting in the formation of a linear polymeric chain running along the [1 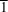 1] direction, with metal–metal distances of 18.0485 (13) Å.

## 3. Supra­molecular features

The water mol­ecules present in **I** form a branched network of hydrogen bonds (Table 2 ▸). Because of the low occupancy and disordering of the O6*W* mol­ecule, its participation in the hydrogen-bonding inter­actions is not considered in further discussion.

**Figure 2:**
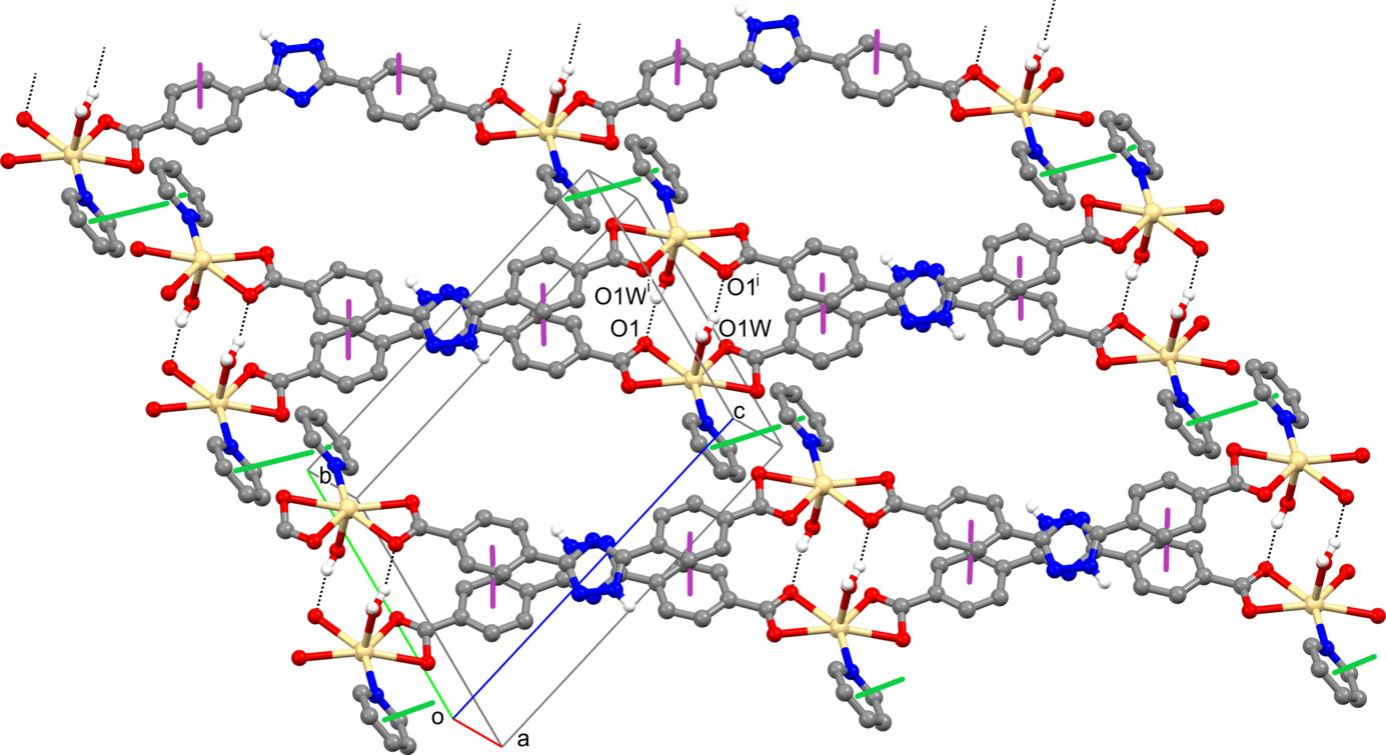
Fragment of the extended sheet in **I** lying parallel to the ( 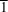 01) plane. C-bound H atoms, N2 pyridine rings, water mol­ecules of crystallization and minor occupancy components *B* of the disordered carboxyl­ate groups have been omitted for clarity. Hydrogen-bonding inter­actions are shown as black dotted lines, π–π stacking inter­actions between benzene rings in double chains and those between coordinated N1 pyridine mol­ecules are shown as lilac and green bold lines, respectively. Symmetry code: (i) –*x* + 1, –*y* + 1, –*z* + 2.

The coordinated water mol­ecule O1*W* plays a specific role in the supra­molecular organization of the crystal of **I**. In particular, acting as proton donors, these mol­ecules of each polymeric chain strongly inter­act with the O1 atoms of the coordinated carboxyl­ate groups of a neighboring one, resulting in the formation of double chains with a Cd1⋯Cd1 distance of 5.425 (7) Å (Fig. 2 ▸). The inter­action between the chains in the dimers is further reinforced by a π–π stacking inter­action between the coaxial and nearly parallel benzene fragments of the anions belonging to different chains with a centroid–centroid distance of 3.667 (1) Å (lilac bold lines in Fig. 2 ▸). Additionally, the coordinated N1 pyridine mol­ecules of each dimeric chain participate in π–π stacking inter­actions [centroid–centroid distance of 3.606 (1) Å] with analogous mol­ecules belonging to neighboring chains (green bold lines in Fig. 2 ▸), resulting in the formation of sheets oriented parallel to the ( 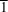 01) plane.

**Figure 3:**
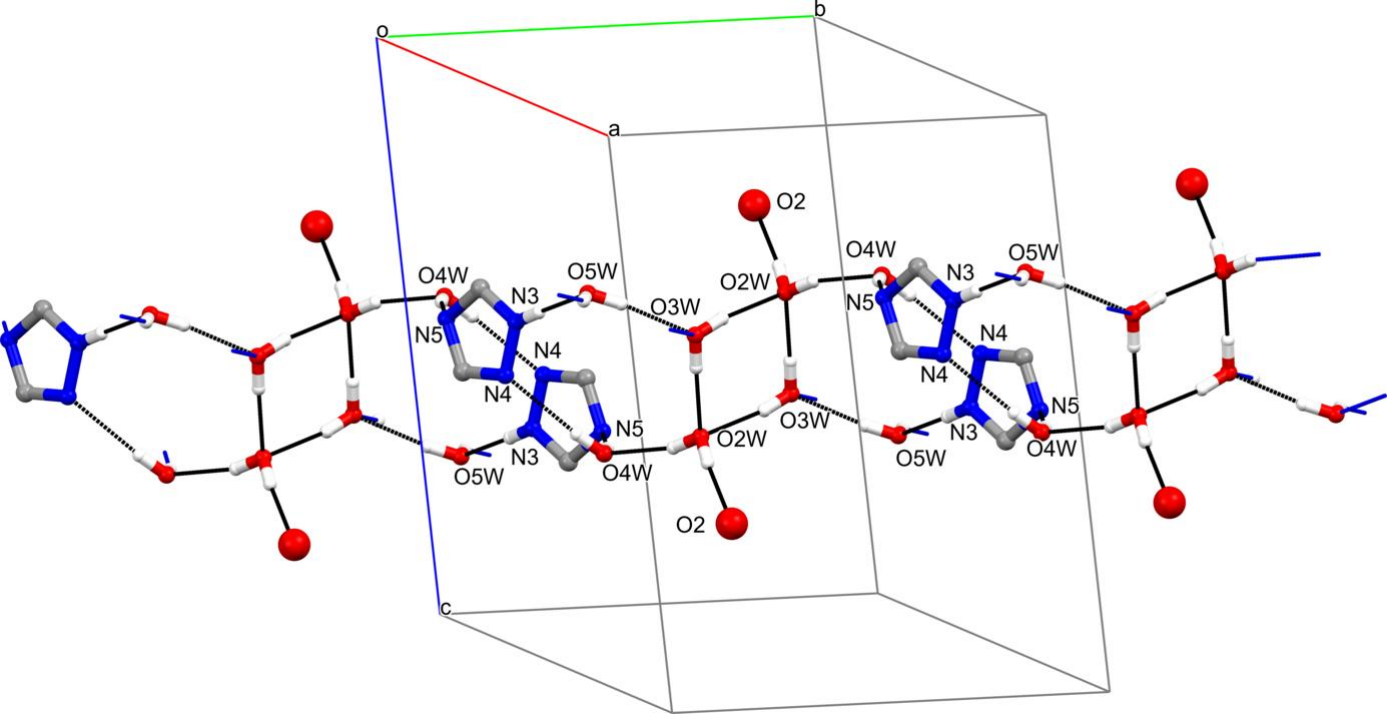
Fragment of the sheet in **I** lying parallel to the (001) plane formed due to hydrogen-bonding inter­actions with the participation of water mol­ecules of crystallization, triazole rings and the non-coordinated O2 atom of the carboxyl­ate groups. Expanded and hanging contacts are shown as black and blue dashed lines, respectively.

The water mol­ecules of crystallization in **I** form hydrogen bonds with the non-coordinated O2 atoms of the carb­oxy­lic groups, the N atoms of the triazole rings, as well as with other water mol­ecules (Table 2 ▸). They all act as the two-proton donors; two of them (O2*W* and O3*W*) function as two-proton acceptors, while O4*W* and O5*W* are single proton acceptors. Inter­estingly, all three nitrogen atoms of the triazole fragment participate in the formation of the hydrogen bonds: N3 as a proton donor and N4 and N5 as proton acceptors. All these inter­actions lead to the arrangement of the above-mentioned constituents into layers lying parallel to the (001) plane (Fig. 3 ▸). Since these layers include organic components (carboxyl­ate groups and triazole fragment) that belong to different coordination-polymeric chains, the network of hydrogen bonds provides the three-dimensional coherence of the crystal of **I**.

## 4. Database survey

A search of the Cambridge Structural Database (CSD, version 5.44, last update September 2023; Groom *et al.*, 2016 ▸) indicated that among more than 55 compounds containing the bct^2–^ anion, five complexes are formed by the Cd^II^ ion [CSD refcodes QIRJAE (Yu *et al.*, 2013 ▸); ZIMJAI (Hou *et al.*, 2013 ▸); WESWOJ (Hou *et al.*, 2017 ▸) and XIXLUO and XIXMAV (Zhang *et al.*, 2019 ▸)]. All of them are coordination polymers and in the first two compounds the only bridging ligand is the bct^2–^ anion, while the others contain bi- or tridentate aromatic amines as additional bridges.

Nevertheless, irrespective of whether the additional polydentate ligands are present, in all cases the bct^2–^ dianion binds to three or four Cd^2+^ ions and this situation is clearly different from that observed in **I**, where the carboxyl­ate ligand displays a μ_2_-bridging function. Inter­estingly, the presence of a common bridging O atom in the coordination spheres of metal ions in the above-mentioned compounds leads to the formation of dimeric polymeric chains, the structures of which are, to some extent, similar to that observed in **I**, where the dimerization proceeds due to the formation of the hydrogen bonds between chains (*vide supra*).

A search of the CSD gave 19 hits related to the structural characterization of compounds containing a Cd^2+^ ion coord­inated by the donor fragment present in **I**, *i.e*., a water mol­ecule, two pyridine ligands or its derivates and two bidentately coordinated carboxyl­ate groups. All have a penta­gonal–bipyramidal structure and the majority of them (16 hits) are characterized by an O(water)/O(carboxyl­ate) equatorial plane and two *trans*-located pyridine ligands [see, for example, BUYVUM10 (Rodesiler *et al.*, 1985 ▸); XATBEA (Li *et al.*, 2005 ▸); LIGWEE (Bania *et al.*, 2007 ▸); OHEFOY, OHEFUE and OHETEC (Saxena & Thirupathi, 2015 ▸)]. Moreover, among them, two complexes formed by the potentially bridging ligands terephthalate [LAMRUP (Croitor *et al.*, 2017 ▸)] and 1,4-phenyl­enedi­acetate [YASMUB (Lin *et al.*, 2005 ▸)] represent coordination polymers. On the other hand, only three among 19 compounds are characterized by a *cis* arrangement of the pyridine ligands. Two of them are cyclic dimers formed by two Cd^II^ ions and two anions of complex bis-oxydi­acetate ligands [NAYFAW (Nath & Baruah, 2012 ▸) and NOLCAU (Nath & Baruah, 2014 ▸)], while the third is a mol­ecular complex that includes two anions of 4-cyano­benzoate [TILCAT (Li *et al.*, 2007 ▸)] and from the point of view of the structural parameters it is the closest structural analogue of **I**. Inter­estingly, in this compound the hydrogen-bonding inter­actions between the coordinated water mol­ecules and O atoms of the coordinated carboxyl­ate groups result in the formation of dimers with a metal-to-metal distance of 5.182 Å, which is close to 5.425 (7) Å observed in **I**.

## 5. Synthesis and crystallization

All chemicals and solvents used in this work were purchased from Sigma–Aldrich and used without further purification. The acid H_2_bct was synthesized according to a procedure described previously (Lopyrev *et al.*, 1977 ▸). For the preparation of the title compound, a solution of CdCl_2_ (28 mg, 0.15 mmol) in water (2 ml) was layered with a solution of 31 mg (0.1 mmol) H_2_bct in 5 ml DMF/py (4:1 by volume). A white precipitate, which had formed over several days, was filtered off, washed with small amounts of DMF and diethyl ether, and dried in air (yield: 24 mg, 35% based on the acid). Analysis calculated (%) for C_26_H_30_CdN_5_O_9.5_: C 46.13, H 4.47, N 10.34; found: C 45.97, H 4.68, N 10.18. Single crystals of **I** suitable for X-ray diffraction analysis were selected from the sample resulting from the synthesis.

## 6. Refinement

Crystal data, data collection and structure refinement details are summarized in Table 3 ▸. The ring H atoms in **I** were placed in geometrically idealized positions and constrained to ride on their parent atoms with a C—H distance of 0.93 Å with *U*_iso_(H) = 1.2*U*_eq_(N). Water H atoms were positioned geometrically (O—H = 0.79–0.85 Å) and refined as riding with *U*_iso_(H) = 1.5*U*_eq_(O). One carboxyl­ate group of the anion (C26/O3/O4) is disordered over two positions with half-occupancy and these components were refined in an isotropic approximation. The water mol­ecule O6*W* is also disordered over two positions with the site occupancies being 0.25. Disordered fragments were modeled using the RESI routine available in *SHELXL*.

## Supplementary Material

Crystal structure: contains datablock(s) I. DOI: 10.1107/S2056989024000185/hb8090sup1.cif

Structure factors: contains datablock(s) I. DOI: 10.1107/S2056989024000185/hb8090Isup2.hkl

CCDC reference: 2321203

Additional supporting information:  crystallographic information; 3D view; checkCIF report

## Figures and Tables

**Table 1:** Selected geometric parameters (Å, °)

Cd1—O1	2.366 (3)	Cd1—O2	2.521 (3)
Cd1—O3*A* ^i^	2.471 (10)	Cd1—O4*A* ^i^	2.538 (6)
Cd1—O3*B* ^i^	2.588 (10)	Cd1—O4*B* ^i^	2.216 (6)
Cd1—N1	2.334 (3)	Cd1—N2	2.340 (3)
Cd1—O1*W*	2.300 (3)		
			
O1*W*—Cd1—N1	88.03 (12)	O1*W*—Cd1—N2	172.97 (14)
O1*W*—Cd1—O1	87.97 (13)	O1*W*—Cd1—O2	92.10 (13)
O1*W*—Cd1—O3*A* ^i^	93.9 (2)	O1*W*—Cd1—O3*B* ^i^	86.8 (2)
O1*W*—Cd1—O4*A* ^i^	98.35 (18)	O1*W*—Cd1—O4*B* ^i^	83.36 (19)
N1—Cd1—N2	93.18 (12)	O1—Cd1—O2	53.22 (8)
O3*A* ^i^—Cd1—O4*A* ^i^	54.05 (13)	O3*B* ^i^—Cd1—O4*B* ^i^	53.15 (14)

Symmetry code: (i) 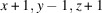 .

**Table 2:** Hydrogen-bond geometry (Å, °)

*D*—H⋯*A*	*D*—H	H⋯*A*	*D*⋯*A*	*D*—H⋯*A*
O1*W*—H1*WB*⋯O1^i^	0.86	1.85	2.694 (4)	166
O2*W*—H2*WA*⋯O4*W* ^ii^	0.85	1.89	2.712 (4)	164
O2*W*—H2*WB*⋯O2	0.85	1.92	2.767 (4)	173
O3*W*—H3*WA*⋯O2*W*	0.85	1.96	2.803 (4)	172
O3*W*—H3*WB*⋯O2*W* ^ii^	0.85	1.96	2.804 (4)	170
O4*W*—H4*WA*⋯N4^iii^	0.85	2.25	3.079 (4)	165
O4*W*—H4*WB*⋯N5	0.85	2.03	2.877 (4)	171
O5*W*—H5*WA*⋯O3*W* ^iv^	0.79	2.12	2.878 (4)	161
O5*W*—H5*WB*⋯O3*W* ^v^	0.85	1.97	2.800 (4)	164
N3—H3⋯O5*W*	0.83 (5)	1.89 (5)	2.720 (4)	177 (5)

Symmetry codes: (i) 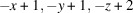 ; (ii) 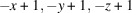 ; (iii) 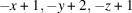 ; (iv) 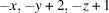 ; (v) 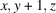 .

**Table 3:** Experimental details

Crystal data	
Chemical formula	[Cd(C_16_H_9_N_3_O_4_)(C_5_H_5_N)_2_(H_2_O)]·4.5H_2_O
*M*_r_	676.95
Crystal system, space group	Triclinic, *P* 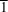
Temperature (K)	293
*a*, *b*, *c* (Å)	8.1674 (5), 12.3033 (6), 15.4877 (8)
α, β, γ (°)	75.226 (5), 86.412 (4), 75.346 (5)
*V* (Å^3^)	1455.89 (14)
*Z*	2
Radiation type	Mo *K*α
μ (mm^−1^)	0.81
Crystal size (mm)	0.45 × 0.03 × 0.03

Data collection	
Diffractometer	Rigaku Xcalibur Eos
Absorption correction	Multi-scan (*CrysAlis PRO*; Rigaku OD, 2022 ▸)
*T*_min_, *T*_max_	0.850, 1.000
No. of measured, independent and observed [*I* > 2σ(*I*)] reflections	14750, 5963, 4538
*R*_int_	0.047
(sin θ/λ)_max_ (Å^−1^)	0.625

Refinement	
*R*[*F* ^2^ > 2σ(*F* ^2^)], *wR*(*F* ^2^), *S*	0.052, 0.099, 1.02
No. of reflections	5963
No. of parameters	378
No. of restraints	6
H-atom treatment	H atoms treated by a mixture of independent and constrained refinement
Δρ_max_, Δρ_min_ (e Å^−3^)	0.80, −0.71

Computer programs: *CrysAlis PRO* (Rigaku OD, 2022 ▸), *SHELXT2018/2* (Sheldrick, 2015*a* ▸), *SHELXL2018/3* (Sheldrick, 2015*b* ▸), *Mercury* (Macrae *et al.*, 2020 ▸) and *publCIF* (Westrip, 2010 ▸).

## supplementary crystallographic information

### Crystal data

**Table:**

[Cd(C_16_H_9_N_3_O_4_)(C_5_H_5_N)_2_(H_2_O)]·4.5H_2_O	*Z* = 2
*M**_r_* = 676.95	*F*(000) = 690
Triclinic, *P*1	*D*_x_ = 1.544 Mg m^−^^3^
*a* = 8.1674 (5) Å	Mo *K*α radiation, λ = 0.71073 Å
*b* = 12.3033 (6) Å	Cell parameters from 4044 reflections
*c* = 15.4877 (8) Å	θ = 1.8–26.4°
α = 75.226 (5)°	µ = 0.81 mm^−^^1^
β = 86.412 (4)°	*T* = 293 K
γ = 75.346 (5)°	Prism, clear light colourless
*V* = 1455.89 (14) Å^3^	0.45 × 0.03 × 0.03 mm

### Data collection

**Table:**

Rigaku Xcalibur Eos diffractometer	5963 independent reflections
Radiation source: fine-focus sealed X-ray tube, Enhance (Mo) X-ray Source	4538 reflections with *I* > 2σ(*I*)
Graphite monochromator	*R*_int_ = 0.047
Detector resolution: 16.1593 pixels mm^-1^	θ_max_ = 26.4°, θ_min_ = 1.9°
ω scans	*h* = −10→10
Absorption correction: multi-scan (CrysAlisPro; Rigaku OD, 2022)	*k* = −15→15
*T*_min_ = 0.850, *T*_max_ = 1.000	*l* = −19→19
14750 measured reflections	

### Refinement

**Table:**

Refinement on *F*^2^	Primary atom site location: dual
Least-squares matrix: full	Hydrogen site location: mixed
*R*[*F*^2^ > 2σ(*F*^2^)] = 0.052	H atoms treated by a mixture of independent and constrained refinement
*wR*(*F*^2^) = 0.099	*w* = 1/[σ^2^(*F*_o_^2^) + (0.0366*P*)^2^ + 0.076*P*] where *P* = (*F*_o_^2^ + 2*F*_c_^2^)/3
*S* = 1.02	(Δ/σ)_max_ < 0.001
5963 reflections	Δρ_max_ = 0.80 e Å^−^^3^
378 parameters	Δρ_min_ = −0.70 e Å^−^^3^
6 restraints	

### Special details

**Table:**

Geometry. All esds (except the esd in the dihedral angle between two l.s. planes) are estimated using the full covariance matrix. The cell esds are taken into account individually in the estimation of esds in distances, angles and torsion angles; correlations between esds in cell parameters are only used when they are defined by crystal symmetry. An approximate (isotropic) treatment of cell esds is used for estimating esds involving l.s. planes.

### Fractional atomic coordinates and isotropic or equivalent isotropic displacement parameters (Å^2^)

**Table:**

	*x*	*y*	*z*	*U*_iso_*/*U*_eq_	Occ. (<1)
Cd1	0.64074 (4)	0.33051 (3)	0.91578 (2)	0.03626 (11)	
O1W	0.4111 (5)	0.3603 (4)	1.0083 (2)	0.1047 (16)	
H1WA	0.313054	0.349393	1.014089	0.157*	
H1WB	0.419894	0.382793	1.055479	0.157*	
O2	0.4683 (4)	0.4504 (2)	0.77939 (17)	0.0454 (7)	
O2W	0.5031 (4)	0.3838 (2)	0.62018 (18)	0.0518 (8)	
H2WA	0.520004	0.310406	0.633938	0.078*	
H2WB	0.483954	0.402446	0.669698	0.078*	
O1	0.5848 (4)	0.5341 (2)	0.85969 (17)	0.0465 (8)	
O4A	−0.1584 (8)	1.1735 (5)	0.0270 (4)	0.0499 (11)*	0.5
O4B	−0.2360 (7)	1.2083 (5)	0.0386 (4)	0.0499 (11)*	0.5
O3W	0.2593 (4)	0.5195 (3)	0.49121 (19)	0.0542 (8)	
H3WA	0.326207	0.479856	0.534058	0.081*	
H3WB	0.320787	0.553956	0.453168	0.081*	
O3B	−0.1896 (12)	1.3767 (5)	0.0317 (5)	0.0563 (12)*	0.5
O3A	−0.1638 (12)	1.3622 (4)	0.0171 (5)	0.0563 (12)*	0.5
O4W	0.4914 (4)	0.8427 (3)	0.3594 (2)	0.0652 (9)	
H4WA	0.574823	0.858316	0.379584	0.098*	
H4WB	0.405463	0.877186	0.384414	0.098*	
O5W	0.0700 (4)	1.3812 (2)	0.44619 (18)	0.0569 (9)	
H5WA	−0.007980	1.408909	0.472957	0.085*	
H5WB	0.141350	1.418159	0.452907	0.085*	
O6WB	0.107 (2)	0.4481 (17)	0.9711 (12)	0.060 (6)*	0.25
H6WA	0.113338	0.516203	0.970506	0.091*	0.25
H6WB	0.010258	0.439113	0.989916	0.091*	0.25
O6WA	0.139 (2)	0.4433 (15)	0.9539 (11)	0.043 (5)*	0.25
H6WC	0.095554	0.514506	0.953184	0.064*	0.25
H6WD	0.069634	0.408256	0.985854	0.064*	0.25
N1	0.5552 (5)	0.1728 (3)	0.8928 (2)	0.0443 (9)	
N2	0.8545 (4)	0.3186 (3)	0.8082 (2)	0.0412 (8)	
N3	0.1923 (5)	1.1490 (3)	0.4659 (2)	0.0424 (9)	
H3	0.157 (6)	1.221 (4)	0.458 (3)	0.074 (18)*	
N4	0.2590 (4)	1.0777 (3)	0.5443 (2)	0.0417 (9)	
N5	0.2250 (4)	0.9751 (3)	0.4486 (2)	0.0379 (8)	
C1	0.3958 (7)	0.1841 (4)	0.8749 (3)	0.0740 (17)	
H1	0.322541	0.257394	0.866349	0.089*	
C2	0.3310 (9)	0.0941 (6)	0.8679 (4)	0.093 (2)	
H2	0.216205	0.105768	0.857408	0.111*	
C3	0.4382 (9)	−0.0124 (5)	0.8767 (3)	0.0694 (17)	
H3A	0.398336	−0.074680	0.870963	0.083*	
C4	0.6026 (8)	−0.0266 (4)	0.8939 (3)	0.0706 (16)	
H4	0.678469	−0.098651	0.899798	0.085*	
C5	0.6579 (7)	0.0691 (4)	0.9029 (3)	0.0602 (14)	
H5	0.771008	0.058824	0.916337	0.072*	
C6	0.8673 (6)	0.2579 (4)	0.7471 (3)	0.0479 (11)	
H6	0.792523	0.211298	0.750025	0.057*	
C7	0.9838 (7)	0.2607 (4)	0.6807 (3)	0.0602 (14)	
H7	0.987319	0.217255	0.639187	0.072*	
C8	1.0954 (6)	0.3276 (4)	0.6753 (3)	0.0627 (14)	
H8	1.177391	0.329792	0.630844	0.075*	
C9	1.0841 (6)	0.3916 (4)	0.7371 (3)	0.0605 (13)	
H9	1.157265	0.439178	0.734878	0.073*	
C10	0.9630 (6)	0.3840 (4)	0.8021 (3)	0.0532 (12)	
H10	0.956621	0.427068	0.844150	0.064*	
C11	0.5034 (5)	0.5385 (4)	0.7923 (2)	0.0368 (10)	
C12	0.4477 (5)	0.6532 (3)	0.7247 (2)	0.0336 (9)	
C13	0.3618 (6)	0.6598 (4)	0.6490 (3)	0.0480 (12)	
H13	0.338297	0.593176	0.640321	0.058*	
C14	0.3102 (6)	0.7635 (4)	0.5858 (3)	0.0471 (11)	
H14	0.254292	0.765596	0.534647	0.057*	
C15	0.3407 (5)	0.8642 (3)	0.5976 (2)	0.0368 (10)	
C16	0.4270 (6)	0.8581 (4)	0.6736 (3)	0.0445 (11)	
H16	0.449859	0.924632	0.682696	0.053*	
C17	0.4793 (5)	0.7534 (4)	0.7362 (3)	0.0417 (10)	
H17	0.536932	0.750681	0.786919	0.050*	
C18	0.2774 (5)	0.9730 (3)	0.5311 (2)	0.0360 (10)	
C19	0.1713 (5)	1.0868 (3)	0.4092 (2)	0.0346 (9)	
C20	0.0987 (5)	1.1348 (3)	0.3197 (2)	0.0344 (9)	
C21	0.0451 (6)	1.0618 (4)	0.2790 (3)	0.0478 (11)	
H21	0.060249	0.983834	0.308029	0.057*	
C22	−0.0295 (6)	1.1028 (4)	0.1969 (3)	0.0541 (13)	
H22	−0.064227	1.052054	0.170832	0.065*	
C23	−0.0546 (5)	1.2180 (4)	0.1514 (3)	0.0436 (11)	
C24	0.0025 (6)	1.2910 (4)	0.1910 (3)	0.0508 (12)	
H24	−0.010726	1.368636	0.161499	0.061*	
C25	0.0788 (6)	1.2489 (4)	0.2741 (3)	0.0519 (12)	
H25	0.117281	1.298581	0.299591	0.062*	
C26	−0.1447 (6)	1.2612 (3)	0.0646 (3)	0.0610 (14)	

### Atomic displacement parameters (Å^2^)

**Table:**

	*U*^11^	*U*^22^	*U*^33^	*U*^12^	*U*^13^	*U*^23^
Cd1	0.0489 (2)	0.03543 (19)	0.02777 (17)	−0.01675 (15)	−0.00309 (13)	−0.00652 (12)
O1W	0.113 (3)	0.178 (4)	0.091 (3)	−0.104 (3)	0.064 (2)	−0.102 (3)
O2	0.062 (2)	0.0361 (17)	0.0383 (16)	−0.0176 (15)	−0.0097 (14)	−0.0012 (13)
O2W	0.063 (2)	0.0454 (19)	0.0480 (18)	−0.0131 (16)	−0.0013 (16)	−0.0126 (15)
O1	0.065 (2)	0.0382 (17)	0.0318 (15)	−0.0016 (15)	−0.0174 (15)	−0.0081 (13)
O3W	0.046 (2)	0.061 (2)	0.0555 (19)	−0.0169 (16)	−0.0044 (15)	−0.0104 (16)
O4W	0.075 (2)	0.048 (2)	0.075 (2)	−0.0079 (18)	0.0133 (19)	−0.0310 (18)
O5W	0.072 (2)	0.0369 (19)	0.063 (2)	−0.0028 (16)	−0.0130 (17)	−0.0209 (16)
N1	0.064 (3)	0.037 (2)	0.0347 (19)	−0.023 (2)	−0.0113 (18)	−0.0010 (16)
N2	0.046 (2)	0.039 (2)	0.039 (2)	−0.0142 (18)	0.0006 (17)	−0.0057 (17)
N3	0.060 (3)	0.025 (2)	0.034 (2)	0.0077 (19)	−0.0170 (17)	−0.0070 (16)
N4	0.055 (2)	0.033 (2)	0.0308 (18)	0.0037 (18)	−0.0132 (16)	−0.0083 (15)
N5	0.045 (2)	0.032 (2)	0.0330 (19)	0.0008 (16)	−0.0100 (16)	−0.0099 (16)
C1	0.080 (4)	0.045 (3)	0.093 (4)	−0.021 (3)	−0.051 (3)	0.006 (3)
C2	0.102 (5)	0.077 (5)	0.107 (5)	−0.045 (4)	−0.057 (4)	−0.001 (4)
C3	0.113 (5)	0.063 (4)	0.050 (3)	−0.051 (4)	0.000 (3)	−0.016 (3)
C4	0.098 (5)	0.046 (3)	0.076 (4)	−0.029 (3)	0.033 (3)	−0.024 (3)
C5	0.068 (4)	0.052 (3)	0.069 (3)	−0.028 (3)	0.021 (3)	−0.022 (3)
C6	0.060 (3)	0.048 (3)	0.041 (3)	−0.023 (2)	0.007 (2)	−0.011 (2)
C7	0.074 (4)	0.058 (3)	0.049 (3)	−0.019 (3)	0.014 (3)	−0.015 (2)
C8	0.056 (3)	0.065 (4)	0.050 (3)	−0.007 (3)	0.010 (3)	0.006 (3)
C9	0.044 (3)	0.067 (4)	0.067 (3)	−0.027 (3)	−0.002 (3)	0.003 (3)
C10	0.055 (3)	0.056 (3)	0.053 (3)	−0.023 (3)	−0.004 (2)	−0.011 (2)
C11	0.036 (2)	0.041 (3)	0.030 (2)	−0.003 (2)	0.0027 (18)	−0.0099 (19)
C12	0.032 (2)	0.036 (2)	0.031 (2)	−0.0028 (19)	−0.0030 (17)	−0.0096 (18)
C13	0.067 (3)	0.037 (3)	0.042 (3)	−0.010 (2)	−0.018 (2)	−0.013 (2)
C14	0.061 (3)	0.041 (3)	0.038 (2)	−0.008 (2)	−0.025 (2)	−0.005 (2)
C15	0.041 (3)	0.031 (2)	0.033 (2)	0.0023 (19)	−0.0064 (18)	−0.0067 (18)
C16	0.058 (3)	0.035 (3)	0.042 (2)	−0.006 (2)	−0.013 (2)	−0.013 (2)
C17	0.050 (3)	0.039 (3)	0.035 (2)	−0.005 (2)	−0.014 (2)	−0.011 (2)
C18	0.039 (2)	0.033 (2)	0.031 (2)	0.0001 (19)	−0.0046 (18)	−0.0079 (18)
C19	0.037 (2)	0.035 (2)	0.030 (2)	−0.0023 (19)	−0.0053 (18)	−0.0101 (18)
C20	0.037 (2)	0.038 (3)	0.026 (2)	−0.0028 (19)	−0.0077 (17)	−0.0077 (18)
C21	0.058 (3)	0.048 (3)	0.043 (3)	−0.020 (2)	−0.009 (2)	−0.010 (2)
C22	0.069 (3)	0.066 (3)	0.037 (3)	−0.037 (3)	−0.017 (2)	−0.009 (2)
C23	0.043 (3)	0.062 (3)	0.029 (2)	−0.024 (2)	−0.0029 (19)	−0.005 (2)
C24	0.067 (3)	0.043 (3)	0.036 (2)	−0.008 (2)	−0.019 (2)	0.003 (2)
C25	0.075 (4)	0.042 (3)	0.039 (3)	−0.011 (3)	−0.023 (2)	−0.008 (2)
C26	0.056 (3)	0.099 (4)	0.042 (3)	−0.051 (3)	−0.002 (2)	−0.011 (3)

### Geometric parameters (Å, º)

**Table:**

Cd1—O1W	2.300 (3)	C2—H2	0.9300
Cd1—O2	2.521 (3)	C2—C3	1.359 (8)
Cd1—O1	2.366 (3)	C3—H3A	0.9300
Cd1—O4A^i^	2.538 (6)	C3—C4	1.345 (7)
Cd1—O4B^i^	2.216 (6)	C4—H4	0.9300
Cd1—O3B^i^	2.588 (10)	C4—C5	1.403 (6)
Cd1—O3A^i^	2.471 (10)	C5—H5	0.9300
Cd1—N1	2.334 (3)	C6—H6	0.9300
Cd1—N2	2.340 (3)	C6—C7	1.356 (6)
O1W—H1WA	0.8401	C7—H7	0.9300
O1W—H1WB	0.8579	C7—C8	1.360 (6)
O2—C11	1.254 (4)	C8—H8	0.9300
O2W—H2WA	0.8498	C8—C9	1.372 (6)
O2W—H2WB	0.8496	C9—H9	0.9300
O1—C11	1.255 (4)	C9—C10	1.369 (6)
O4A—C26	1.379 (5)	C10—H10	0.9300
O4B—C26	1.2476 (10)	C11—C12	1.509 (5)
O3W—H3WA	0.8498	C12—C13	1.378 (5)
O3W—H3WB	0.8500	C12—C17	1.377 (5)
O3B—C26	1.343 (6)	C13—H13	0.9300
O3A—C26	1.2485 (10)	C13—C14	1.381 (5)
O4W—H4WA	0.8500	C14—H14	0.9300
O4W—H4WB	0.8501	C14—C15	1.381 (5)
O5W—H5WA	0.7895	C15—C16	1.386 (5)
O5W—H5WB	0.8503	C15—C18	1.461 (5)
O6WB—H6WA	0.8499	C16—H16	0.9300
O6WB—H6WB	0.8497	C16—C17	1.384 (5)
O6WA—H6WC	0.8547	C17—H17	0.9300
O6WA—H6WD	0.8535	C19—C20	1.459 (5)
N1—C1	1.314 (6)	C20—C21	1.385 (5)
N1—C5	1.315 (6)	C20—C25	1.374 (5)
N2—C6	1.332 (5)	C21—H21	0.9300
N2—C10	1.324 (5)	C21—C22	1.363 (5)
N3—H3	0.83 (5)	C22—H22	0.9300
N3—N4	1.355 (4)	C22—C23	1.382 (6)
N3—C19	1.347 (5)	C23—C24	1.389 (5)
N4—C18	1.325 (5)	C23—C26	1.481 (5)
N5—C18	1.364 (4)	C24—H24	0.9300
N5—C19	1.324 (5)	C24—C25	1.382 (5)
C1—H1	0.9300	C25—H25	0.9300
C1—C2	1.372 (6)		
			
O1W—Cd1—O2	92.10 (13)	C5—C4—H4	120.6
O1W—Cd1—O1	87.97 (13)	N1—C5—C4	122.3 (5)
O1W—Cd1—O4A^i^	98.35 (18)	N1—C5—H5	118.9
O1W—Cd1—O3B^i^	86.8 (2)	C4—C5—H5	118.9
O1W—Cd1—O3A^i^	93.9 (2)	N2—C6—H6	118.3
O1W—Cd1—N1	88.03 (12)	N2—C6—C7	123.4 (4)
O1W—Cd1—N2	172.97 (14)	C7—C6—H6	118.3
O2—Cd1—O4A^i^	164.94 (13)	C6—C7—H7	120.3
O2—Cd1—O3B^i^	134.39 (13)	C6—C7—C8	119.5 (4)
O1—Cd1—O2	53.22 (8)	C8—C7—H7	120.3
O1—Cd1—O4A^i^	137.54 (13)	C7—C8—H8	120.8
O1—Cd1—O3B^i^	81.19 (13)	C7—C8—C9	118.3 (4)
O1—Cd1—O3A^i^	83.77 (11)	C9—C8—H8	120.8
O4B^i^—Cd1—O1W	83.36 (19)	C8—C9—H9	120.7
O4B^i^—Cd1—O2	171.16 (12)	C10—C9—C8	118.7 (4)
O4B^i^—Cd1—O1	133.80 (12)	C10—C9—H9	120.7
O4B^i^—Cd1—O4A^i^	15.3 (2)	N2—C10—C9	123.4 (4)
O4B^i^—Cd1—O3B^i^	53.15 (14)	N2—C10—H10	118.3
O4B^i^—Cd1—O3A^i^	51.93 (13)	C9—C10—H10	118.3
O4B^i^—Cd1—N1	85.58 (13)	O2—C11—O1	121.9 (4)
O4B^i^—Cd1—N2	103.63 (18)	O2—C11—C12	119.3 (3)
O3A^i^—Cd1—O2	136.29 (11)	O1—C11—C12	118.8 (3)
N1—Cd1—O2	86.69 (10)	C13—C12—C11	119.9 (3)
N1—Cd1—O1	139.50 (11)	C17—C12—C11	121.9 (3)
N1—Cd1—O4A^i^	82.87 (14)	C17—C12—C13	118.2 (4)
N1—Cd1—O3B^i^	138.73 (14)	C12—C13—H13	119.4
N1—Cd1—O3A^i^	136.72 (13)	C12—C13—C14	121.1 (4)
N1—Cd1—N2	93.18 (12)	C14—C13—H13	119.4
N2—Cd1—O2	81.06 (10)	C13—C14—H14	119.6
N2—Cd1—O1	86.61 (11)	C13—C14—C15	120.7 (4)
N2—Cd1—O4A^i^	88.68 (16)	C15—C14—H14	119.6
N2—Cd1—O3B^i^	96.7 (2)	C14—C15—C16	118.4 (4)
N2—Cd1—O3A^i^	89.9 (2)	C14—C15—C18	119.0 (3)
Cd1—O1W—H1WA	138.5	C16—C15—C18	122.7 (4)
Cd1—O1W—H1WB	119.7	C15—C16—H16	119.8
H1WA—O1W—H1WB	101.4	C17—C16—C15	120.4 (4)
C11—O2—Cd1	88.7 (2)	C17—C16—H16	119.8
H2WA—O2W—H2WB	104.5	C12—C17—C16	121.2 (4)
C11—O1—Cd1	95.9 (2)	C12—C17—H17	119.4
C26—O4A—Cd1^ii^	86.0 (3)	C16—C17—H17	119.4
C26—O4B—Cd1^ii^	104.5 (4)	N4—C18—N5	113.3 (3)
H3WA—O3W—H3WB	104.5	N4—C18—C15	124.8 (3)
C26—O3B—Cd1^ii^	84.7 (4)	N5—C18—C15	121.8 (3)
C26—O3A—Cd1^ii^	91.8 (5)	N3—C19—C20	125.6 (4)
H4WA—O4W—H4WB	104.5	N5—C19—N3	108.8 (3)
H5WA—O5W—H5WB	101.0	N5—C19—C20	125.7 (3)
H6WA—O6WB—H6WB	109.5	C21—C20—C19	118.3 (4)
H6WC—O6WA—H6WD	103.5	C25—C20—C19	123.5 (3)
C1—N1—Cd1	119.7 (3)	C25—C20—C21	118.2 (4)
C1—N1—C5	117.4 (4)	C20—C21—H21	119.6
C5—N1—Cd1	122.8 (3)	C22—C21—C20	120.9 (4)
C6—N2—Cd1	124.7 (3)	C22—C21—H21	119.6
C10—N2—Cd1	118.3 (3)	C21—C22—H22	119.3
C10—N2—C6	116.8 (4)	C21—C22—C23	121.5 (4)
N4—N3—H3	123 (3)	C23—C22—H22	119.3
C19—N3—H3	126 (3)	C22—C23—C24	117.9 (4)
C19—N3—N4	110.5 (3)	C22—C23—C26	120.1 (3)
C18—N4—N3	103.0 (3)	C24—C23—C26	122.0 (4)
C19—N5—C18	104.4 (3)	C23—C24—H24	119.8
N1—C1—H1	118.1	C25—C24—C23	120.4 (4)
N1—C1—C2	123.9 (5)	C25—C24—H24	119.8
C2—C1—H1	118.1	C20—C25—C24	121.1 (4)
C1—C2—H2	120.7	C20—C25—H25	119.4
C3—C2—C1	118.5 (6)	C24—C25—H25	119.4
C3—C2—H2	120.7	O4A—C26—C23	113.2 (4)
C2—C3—H3A	120.5	O4B—C26—O3B	114.1 (6)
C4—C3—C2	119.1 (5)	O4B—C26—C23	123.3 (4)
C4—C3—H3A	120.5	O3B—C26—C23	116.7 (5)
C3—C4—H4	120.6	O3A—C26—O4A	119.9 (6)
C3—C4—C5	118.8 (5)	O3A—C26—C23	123.9 (5)
			
Cd1—O2—C11—O1	−4.7 (4)	C10—N2—C6—C7	−0.1 (7)
Cd1—O2—C11—C12	174.7 (3)	C11—C12—C13—C14	−179.6 (4)
Cd1—O1—C11—O2	5.1 (4)	C11—C12—C17—C16	−179.8 (4)
Cd1—O1—C11—C12	−174.4 (3)	C12—C13—C14—C15	−1.2 (7)
Cd1^ii^—O4A—C26—O3A	−28.7 (7)	C13—C12—C17—C16	−0.1 (6)
Cd1^ii^—O4A—C26—C23	170.0 (3)	C13—C14—C15—C16	1.2 (7)
Cd1^ii^—O4B—C26—O3B	20.2 (7)	C13—C14—C15—C18	−177.3 (4)
Cd1^ii^—O4B—C26—C23	172.0 (4)	C14—C15—C16—C17	−0.7 (6)
Cd1^ii^—O3B—C26—O4B	−16.7 (6)	C14—C15—C18—N4	162.0 (4)
Cd1^ii^—O3B—C26—C23	−170.4 (4)	C14—C15—C18—N5	−15.6 (6)
Cd1^ii^—O3A—C26—O4A	29.5 (7)	C15—C16—C17—C12	0.1 (7)
Cd1^ii^—O3A—C26—C23	−171.3 (4)	C16—C15—C18—N4	−16.5 (6)
Cd1—N1—C1—C2	−174.2 (4)	C16—C15—C18—N5	166.0 (4)
Cd1—N1—C5—C4	176.2 (3)	C17—C12—C13—C14	0.6 (7)
Cd1—N2—C6—C7	174.0 (4)	C18—N5—C19—N3	0.6 (4)
Cd1—N2—C10—C9	−174.4 (4)	C18—N5—C19—C20	−178.6 (4)
O2—C11—C12—C13	−1.2 (6)	C18—C15—C16—C17	177.8 (4)
O2—C11—C12—C17	178.6 (4)	C19—N3—N4—C18	0.6 (4)
O1—C11—C12—C13	178.3 (4)	C19—N5—C18—N4	−0.2 (5)
O1—C11—C12—C17	−1.9 (6)	C19—N5—C18—C15	177.6 (4)
N1—C1—C2—C3	−2.6 (9)	C19—C20—C21—C22	177.5 (4)
N2—C6—C7—C8	0.5 (8)	C19—C20—C25—C24	−177.1 (4)
N3—N4—C18—N5	−0.2 (5)	C20—C21—C22—C23	−0.1 (7)
N3—N4—C18—C15	−178.0 (4)	C21—C20—C25—C24	2.0 (7)
N3—C19—C20—C21	−163.4 (4)	C21—C22—C23—C24	1.6 (7)
N3—C19—C20—C25	15.7 (7)	C21—C22—C23—C26	−176.5 (4)
N4—N3—C19—N5	−0.7 (5)	C22—C23—C24—C25	−1.2 (7)
N4—N3—C19—C20	178.4 (4)	C22—C23—C26—O4A	−15.3 (7)
N5—C19—C20—C21	15.5 (6)	C22—C23—C26—O4B	18.0 (8)
N5—C19—C20—C25	−165.3 (4)	C22—C23—C26—O3B	169.1 (6)
C1—N1—C5—C4	0.8 (7)	C22—C23—C26—O3A	−175.7 (7)
C1—C2—C3—C4	1.5 (9)	C23—C24—C25—C20	−0.6 (7)
C2—C3—C4—C5	0.5 (8)	C24—C23—C26—O4A	166.7 (5)
C3—C4—C5—N1	−1.7 (7)	C24—C23—C26—O4B	−160.1 (5)
C5—N1—C1—C2	1.4 (8)	C24—C23—C26—O3B	−8.9 (8)
C6—N2—C10—C9	0.1 (7)	C24—C23—C26—O3A	6.2 (9)
C6—C7—C8—C9	−1.0 (8)	C25—C20—C21—C22	−1.7 (7)
C7—C8—C9—C10	1.0 (7)	C26—C23—C24—C25	176.8 (4)
C8—C9—C10—N2	−0.6 (7)		

Symmetry codes: (i) *x*+1, *y*−1, *z*+1; (ii) *x*−1, *y*+1, *z*−1.

### Hydrogen-bond geometry (Å, º)

**Table:**

*D*—H···*A*	*D*—H	H···*A*	*D*···*A*	*D*—H···*A*
O1*W*—H1*WB*···O1^iii^	0.86	1.85	2.694 (4)	166
O2*W*—H2*WA*···O4*W*^iv^	0.85	1.89	2.712 (4)	164
O2*W*—H2*WB*···O2	0.85	1.92	2.767 (4)	173
O3*W*—H3*WA*···O2*W*	0.85	1.96	2.803 (4)	172
O3*W*—H3*WB*···O2*W*^iv^	0.85	1.96	2.804 (4)	170
O4*W*—H4*WA*···N4^v^	0.85	2.25	3.079 (4)	165
O4*W*—H4*WB*···N5	0.85	2.03	2.877 (4)	171
O5*W*—H5*WA*···O3*W*^vi^	0.79	2.12	2.878 (4)	161
O5*W*—H5*WB*···O3*W*^vii^	0.85	1.97	2.800 (4)	164
N3—H3···O5*W*	0.83 (5)	1.89 (5)	2.720 (4)	177 (5)

Symmetry codes: (iii) −*x*+1, −*y*+1, −*z*+2; (iv) −*x*+1, −*y*+1, −*z*+1; (v) −*x*+1, −*y*+2, −*z*+1; (vi) −*x*, −*y*+2, −*z*+1; (vii) *x*, *y*+1, *z*.

## References

[bb1] Bania, K., Barooah, N. & Baruah, J. B. (2007). *Polyhedron*, **26**, 2612–2620.

[bb2] Chen, B., Yang, Z., Zhu, Y. & Xia, Y. (2014). *J. Mater. Chem. A*, **2**, 16811–16831.

[bb3] Croitor, L., Coropceanu, E. B., Duca, G., Siminel, A. V. & Fonari, M. S. (2017). *Polyhedron*, **129**, 9–21.

[bb4] Gao, Y.-H., Huang, P.-P., Xu, H.-T., Huang, P., Liu, B., Lu, J.-F. & Ge, H.-G. (2023). *J. Mol. Struct.* **1281**, 135106.

[bb5] Groom, C. R., Bruno, I. J., Lightfoot, M. P. & Ward, S. C. (2016). *Acta Cryst.* B**72**, 171–179. 10.1107/S2052520616003954 PMC4822653 27048719

[bb6] Hou, X.-Y., Wang, X., Fu, F., Wang, J.-J. & Tang, L. (2013). *J. Coord. Chem.* **66**, 3126–3136.

[bb7] Hou, X.-Y., Wang, X., Ren, Y.-X., Wang, J.-J., Jin, W., Kang, W.-W., Ma, X. & Han, X.-X. (2017). *Jiegou Huaxue*, **36**, 2067–2072.

[bb8] Kaskel, S. (2016). Editor. *The Chemistry of Metal–Organic Frameworks: Synthesis, Characterization and Applications*. Weinheim: Wiley-VCH.

[bb9] Li, H.-X., Zhang, Z.-H., Fang, H., Xue, D.-X. & Bai, J. (2022). *J. Mater. Chem. A*, **10**, 14795–14798.

[bb10] Li, Y., Li, G.-Q., Zheng, F.-K., Zou, J.-P., Zou, W.-Q., Guo, G.-C., Lu, C.-Z. & Huang, J.-S. (2007). *J. Mol. Struct.* **842**, 38–45.

[bb11] Li, Y., Wu, A.-Q., Zheng, F.-K., Fu, M.-L., Guo, G.-C. & Huang, J.-S. (2005). *Inorg. Chem. Commun.* **8**, 708–712.

[bb12] Lin, X., Wang, Y.-Q., Cao, R., Li, F. & Bi, W.-H. (2005). *Acta Cryst.* C**61**, m292–m294. 10.1107/S0108270105007274 15930669

[bb13] Lopyrev, V. A., Chipanina, N. N., Rozinova, L. G., Sarapulova, G. I., Sultangareev, R. G. & Voronkov, M. G. (1977). *Chem. Heterocycl. Compd.* **13**, 1346–1349.

[bb14] Lu, J. F., Gao, J. H., Tang, B., Sun, M. & Ge, H. G. (2021). *Crystallogr. Rep.* **66**, 1295–1299.

[bb15] Lu, X., Tang, Y., Yang, G. & Wang, Y.-Y. (2023). *CrystEngComm*, **25**, 896–908.

[bb16] Luo, L., Xie, Y., Hou, S.-L., Ma, Y. & Zhao, B. (2022). *Inorg. Chem.* **61**, 9990–9996. 10.1021/acs.inorgchem.2c00850 35715016

[bb17] MacGillivray, L. R. & Lukehart, C. M. (2014). Editors. *Metal–Organic Framework Materials*. Hoboken: John Wiley and Sons.

[bb18] Macrae, C. F., Sovago, I., Cottrell, S. J., Galek, P. T. A., McCabe, P., Pidcock, E., Platings, M., Shields, G. P., Stevens, J. S., Towler, M. & Wood, P. A. (2020). *J. Appl. Cryst.* **53**, 226–235. 10.1107/S1600576719014092 PMC6998782 32047413

[bb19] Nath, B. & Baruah, J. B. (2012). *Dalton Trans.* **41**, 7115–7126. 10.1039/c2dt30554b 22565819

[bb20] Nath, B. & Baruah, J. B. (2014). *Polyhedron*, **79**, 291–299.

[bb21] Rao, C. N. R., Natarajan, S. & Vaidhyanathan, R. (2004). *Angew. Chem. Int. Ed.* **43**, 1466–1496. 10.1002/anie.200300588 15022215

[bb22] Rigaku OD (2022). *CrysAlis PRO*. Rigaku Oxford Diffraction, Yarnton, England.

[bb23] Rodesiler, P. F., Griffith, E. A. H., Charles, N. G. & Amma, E. L. (1985). *Acta Cryst.* C**41**, 673–678.

[bb24] Saxena, P. & Thirupathi, N. (2015). *Polyhedron*, **98**, 238–250.

[bb25] Sheldrick, G. M. (2015*a*). *Acta Cryst.* A**71**, 3–8.

[bb26] Sheldrick, G. M. (2015*b*). *Acta Cryst.* C**71**, 3–8.

[bb27] Sun, X., Gu, J., Yuan, Y., Yu, C., Li, J., Shan, H., Li, G. & Liu, Y. (2019). *Inorg. Chem.* **58**, 7480–7487. 10.1021/acs.inorgchem.9b00701 31074626

[bb28] Tian, X.-R., Shi, Y., Hou, S.-L., Ma, Y. & Zhao, B. (2021). *Inorg. Chem.* **60**, 15383–15389. 10.1021/acs.inorgchem.1c02034 34590842

[bb29] Wang, X., Xu, Q.-W., Wei, M.-M., Chen, J.-Y., Wang, H.-H. & Li, X. (2022). *CrystEngComm*, **24**, 6367–6375.

[bb30] Westrip, S. P. (2010). *J. Appl. Cryst.* **43**, 920–925.

[bb31] Yoshinari, N. & Konno, T. (2023). *Coord. Chem. Rev.* **474**, 214850.

[bb32] Yu, M., Hu, M. & Wu, Z. (2013). *RSC Adv.* **3**, 25175–25183.

[bb33] Zhang, Y.-X., Lin, H., Wen, Y. & Zhu, Q.-L. (2019). *Cryst. Growth Des.* **19**, 1057–1063.

[bb34] Zhao, J., Yuan, J., Fang, Z., Huang, S., Chen, Z., Qiu, F., Lu, C., Zhu, J. & Zhuang, X. (2022). *Coord. Chem. Rev.* **471**, 214735.

